# Supplementary material for: Integrated microcavity electric field sensors using Pound-Drever-Hall detection
Source: Nat Commun. 2024 Feb 15;15:1386. doi: 10.1038/s41467-024-45699-w (PMC10869830; doi:10.1038/s41467-024-45699-w)
Supplement: Supplementary file 1 — Supplementary Information [file 41467_2024_45699_MOESM1_ESM.pdf]

Supplementary Information for "Integrated microcavity electric field sensors using Pound-Drever-Hall detection"

Xinyu Ma,[1] Zhaoyu Cai,[2] Chijie Zhuang,[1,*] Xiangdong Liu,[1] Zhecheng Zhang,[1] Kewei Liu,[2] Bo Cao,[2] Jinliang He,[1] Changxi Yang,[2] Chengying Bao,[2,†] and Rong Zeng[1,‡]
[1]State Key Lab of Power Systems, Department of Electrical Engineering, Tsinghua University, Beijing 100084, China.
[2]State Key Laboratory of Precision Measurement Technology and Instruments, Department of Precision Instruments, Tsinghua University, Beijing 100084, China.
Corresponding authors: *chijie@tsinghua.edu.cn, †cbao@tsinghua.edu.cn, ‡zengrong@tsinghua.edu.cn

1. Derivation of the MEFS sensing signal. Our PDH detection based sensing approach can be understood in the frequency domain. The laser, whose angular frequency is ω_l, is phase modulated at an angular frequency of Ω_m. Considering a relatively weak modulation, it creates sidebands at $\omega_l \pm \Omega_m$. The sidebands and the resonance transmission for the pump are plotted in Fig. S1. Since Ω_m is much larger than the cavity linewidth κ, we assume these sidebands do not enter the microcavity, and only the carrier does. The intracavity field at ω_l will be phase modulated by the sensing electric field at a frequency Ω_s. This electro-optical modulation will also creates intracavity sidebands at frequencies $\omega_l \pm \Omega_s$. The multi-heterodyne between sidebands $\omega_l \pm \Omega_m$ and $\omega_l \pm \Omega_s$ will generate signals at frequencies of $\Omega_m + \Omega_s$ and $\Omega_m - \Omega_s$ (see Fig. S1) on PD3 in Fig. 2a of the main text. These heterodyne signals mix with the local oscillator frequency Ω_m and generate the sensing signal at Ω_s. Hence, to derive the sensing signal, we need to the analyze the sidebands at $\omega_l \pm \Omega_m$ and $\omega_l \pm \Omega_s$.

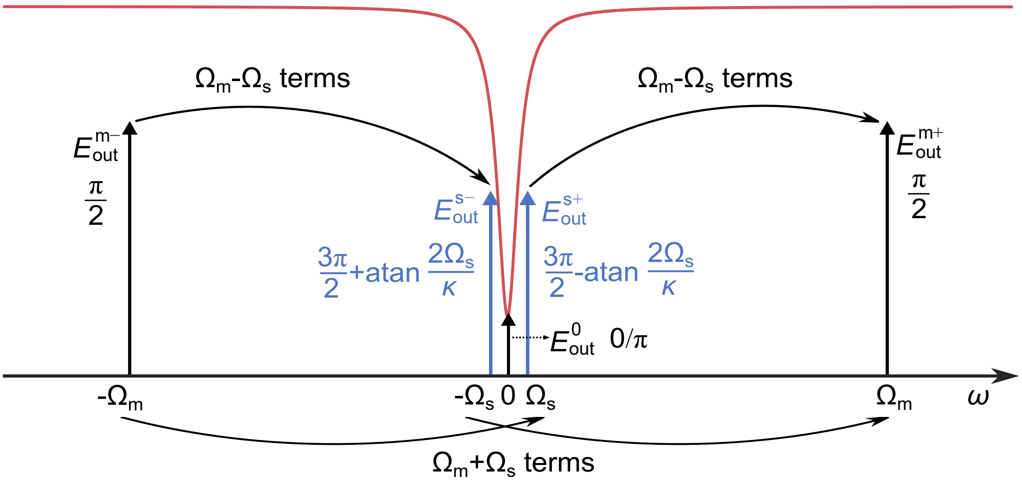

Fig. S1: **The spectral diagram of the output light field from the microcavity.** The red curve is an illustration of the cavity transmission. The sidebands generated by the phase modulation of the pump ($E_{\rm out}^{m\pm}$), and intracavity field ($E_{\rm out}^{s\pm}$), and the additional phase they carry are also illustrated.These beams beat on the PD and generate RF signals with different frequencies, in which the $\Omega_m - \Omega_s$ terms and the $\Omega_m + \Omega_s$ terms result in the sensing signal.

The weakly phase modulated pump can be written as [S1],

$$E_{\rm in}e^{i\omega_l t}e^{i\beta\cos\Omega_m t} \approx E_{\rm in}e^{i\omega_l t}\left[1 + iJ_1(\beta)e^{i\Omega_m t} + iJ_1(\beta)e^{-i\Omega_m t}\right], \tag{S1}$$

where $|E_{\rm in}|^2 = P_{\rm in}$ is the pump power, $J_1(\beta)$ is the 1st order Bessel function of the first kind, β is the amplitude of the phase modulation. Since these sidebands are off-resonance, they can be regarded as passing through the microcavity directly. The output of the sidebands in the waveguide ($E_{\rm out}^{m+}$, $E_{\rm out}^{m-}$) can be written as,

$$E_{\rm out}^{m\pm} = iJ_1(\beta)E_{\rm in}e^{i(\omega_l \pm \Omega_m)t}. \tag{S2}$$

Since the $\omega_l \pm \Omega_s$ sidebands are in the cavity, their amplitude and phase are impacted by the cavity response. The motion equation for the intracavity field A subject to a coherent pump (the carrier at ω_l only) is,

$$\frac{\partial A}{\partial t} = -(\frac{\kappa}{2} - i\Delta\omega - i\delta\omega\cos\Omega_s t)A + i\sqrt{\frac{\kappa_e}{T_{\rm R}}}E_{\rm in}, \tag{S3}$$

where A is the envelope of the intracavity field, t is the slow time, $\Delta\omega = \omega_0 - \omega_l$ is the detuning of pump laser from the resonance ω_0, $\kappa = \kappa_0 + \kappa_e$ is the total loss rate of the cavity including the intrinsic loss rate κ_0 and the external coupling rate κ_e, T_R is the round-trip time, $\delta\omega$ is the amplitude of the resonance shift due to the phase modulation induced by the sensing field ε via the electrode of the microcavity.

This equation can be solved in the frequency domain,

$$\left(\frac{\kappa}{2} + i\omega - i\Delta\omega\right)\tilde{A}(\omega) = i\frac{\delta\omega}{2}\left[\tilde{A}\left(\omega - \Omega_s\right) + \tilde{A}\left(\omega + \Omega_s\right)\right] + i\sqrt{\frac{\kappa_e}{T_R}}E_\mathrm{in}\delta(\omega), \tag{S4}$$

where $\tilde{A}(\omega)$ is the Fourier transform of $A(t)$, ω is a frequency relative to ω_l, and δ is the Dirac delta function.

Since we lock the laser to the resonance in the experiment, $\Delta\omega$=0. We first consider $\omega = 0$ in Eq. (S4), the intracavity $\tilde{A}(0)$ can be solved as (we assume the sensing field is weak and $\delta\omega$ is small),

$$\tilde{A}(0) = \frac{i\sqrt{\kappa_e/T_R}}{\kappa/2}E_\mathrm{in}\ , \tag{S5}$$

The corresponding output field in the waveguide E^0_out is,

$$E^0_\mathrm{out} = \left[E_\mathrm{in} + i\sqrt{\kappa_e T_R}\tilde{A}(0)\right]e^{i\omega_l t} = \left(1 - \frac{2\kappa_e}{\kappa}\right)E_\mathrm{in}e^{i\omega_l t}, \tag{S6}$$

where i corresponds to the $\pi/2$ phase shift when coupling from the cavity to the waveguide.

By setting $\omega = \Omega_s$ and $\omega = -\Omega_s$ in Eq. (S4), the amplitude of the intracavity of the sidebands at $\omega_l \pm \Omega_s$ denoted as A_{s+}, A_{s-} can be derived as,

$$A_{s+} = \tilde{A}(\Omega_s) = \frac{i\delta\omega\tilde{A}(0)}{2\left(\kappa/2 + i\Omega_s\right)}, \tag{S7}$$

$$A_{s-} = \tilde{A}(-\Omega_s) = \frac{i\delta\omega\tilde{A}(0)}{2\left(\kappa/2 - i\Omega_s\right)}. \tag{S8}$$

Then the output fields in the waveguide for these sidebands, denoted as E^{s+}_out and E^{s-}_out, can be written as,

$$E^{s+}_\mathrm{out} = i\sqrt{\kappa_e T_R}\cdot A_{s+}e^{i(\omega_l+\Omega_s)t} = -\frac{\sqrt{\kappa_e T_R}\cdot\delta\omega\tilde{A}(0)}{2\left(\kappa/2 + i\Omega_s\right)}e^{i(\omega_l+\Omega_s)t}, \tag{S9}$$

$$E^{s-}_\mathrm{out} = i\sqrt{\kappa_e T_R}\cdot A_{s-}e^{i(\omega_l-\Omega_s)t} = -\frac{\sqrt{\kappa_e T_R}\cdot\delta\omega\tilde{A}(0)}{2\left(\kappa/2 - i\Omega_s\right)}e^{i(\omega_l-\Omega_s)t}. \tag{S10}$$

Thus, the optical field in the output waveguide is,

$$E_\mathrm{out} = E^{m-}_\mathrm{out} + E^{s-}_\mathrm{out} + E^0_\mathrm{out} + E^{s+}_\mathrm{out} + E^{m+}_\mathrm{out}. \tag{S11}$$

And the output of PD3 is

$$V_\mathrm{PD} = K_\mathrm{PD}|E_\mathrm{out}|^2, \tag{S12}$$

where K_PD is the conversion factor of the PD3.

We are interested in the RF sidebands at $\Omega_m \pm \Omega_s$, which are

$$V^\mathrm{m+s}_\mathrm{PD} = K_\mathrm{PD}J_1(\beta)E_\mathrm{in}\sqrt{\kappa_e T_R}\left[e^{-i(\Omega_m+\Omega_s)t}\left(A^*_{s+} + A_{s-}\right) + e^{i(\Omega_m+\Omega_s)t}\left(A_{s+} + A^*_{s-}\right)\right], \tag{S13}$$

$$V^\mathrm{m-s}_\mathrm{PD} = K_\mathrm{PD}J_1(\beta)E_\mathrm{in}\sqrt{\kappa_e T_R}\left[e^{-i(\Omega_m-\Omega_s)t}\left(A^*_{s-} + A_{s+}\right) + e^{i(\Omega_m-\Omega_s)t}\left(A_{s-} + A^*_{s+}\right)\right]. \tag{S14}$$

These $\Omega_m \pm \Omega_s$ sidebands will mix with the local oscillator at Ω_m to generate the sensing signal at Ω_s after low pass filtering,

$$\begin{aligned} V_{\mathrm{RF}}(t) =& 4J_1(\beta)K_{\mathrm{PD}}K_{\mathrm{mix}}|E_{\mathrm{in}}|^2\frac{\kappa_e}{\kappa}\delta\omega\left[\frac{\kappa/2}{(\kappa/2)^2+\Omega_s^2}\cos\left(\Omega_s t\right)+\frac{\Omega_s}{(\kappa/2)^2+\Omega_s^2}\sin\left(\Omega_s t\right)\right] \\ =& KP_{\mathrm{in}}\frac{\kappa_e}{\kappa}\frac{\varepsilon}{\sqrt{(\kappa/2)^2+\Omega_s^2}}\cos\left[\Omega_s t-\mathrm{atan}\left(\frac{2\Omega_s}{\kappa}\right)\right], \end{aligned} \tag{S15}$$

where $K=4J_1(\beta)K_{\mathrm{PD}}K_{\mathrm{mix}}K_{\mathrm{eo}}$ ($\delta\omega=K_{\mathrm{eo}}\varepsilon$) is a conversion factor including response from the external-cavity phase modulation, photodetector, local oscillator and mixer, and microcavity electro-optical modulation efficiency that is affected by the microcavity electrode and the antenna, respectively; $-\mathrm{atan}\left(2\Omega_{\mathrm{s}}/\kappa\right)$ is a phase resonance caused by the cavity resonance.

The detected RF power of Ω_s is proportional to,

$$\langle|V_{\mathrm{RF}}|^2\rangle=\frac{1}{2}K^2P_{\mathrm{in}}^2\left(\frac{\kappa_e}{\kappa}\right)^2\frac{\varepsilon^2}{(\kappa/2)^2+\Omega_s^2}, \tag{S16}$$

which follows a Lorentzian function with a 3 dB bandwidth of $\kappa/2$ (note that the center frequency of the Lorentzian function is zero). Therefore, high Q-factors improve the sensitivity, but at the expense of a reduced bandwidth. It can be found that for a fixed intrinsic Q-factor (i.e., fixed κ_0), critical coupling ($\kappa_e=\kappa_0$) is the optimal condition to enhance the sensitivity for $\Omega_s \ll \kappa$.

Sensing with strong electric fields.- When considering a relatively strong electric field (large $\delta\omega$), the higher-order intracavity sidebands e.g., $\tilde{A}(\pm 2\Omega_s)$ and depletion of $\tilde{A}(0)$ should be considered when deriving the MEFS response. When considering the second-order sidebands, by substituting $\omega=\Omega_s$ and $\omega=2\Omega_s$ into Eq. S4, we have two equations as ($\tilde{A}(3\Omega_s)$ and higher orders were still omitted),

$$\left(\frac{\kappa}{2}+i\Omega_s\right)\tilde{A}(\Omega_s)=i\frac{\delta\omega}{2}\left[\tilde{A}(0)+\tilde{A}(2\Omega_s)\right], \tag{S17}$$

$$\left(\frac{\kappa}{2}+i2\Omega_s\right)\tilde{A}(2\Omega_s)=i\frac{\delta\omega}{2}\tilde{A}(\Omega_s). \tag{S18}$$

Thus, $\tilde{A}(\Omega_s)$ is derived as,

$$\tilde{A}\left(\Omega_s\right)=\frac{i\delta\omega}{2\left[i\Omega_s+\frac{\kappa}{2}+\frac{\delta\omega^2}{2(i4\Omega_s+\kappa)}\right]}\tilde{A}(0). \tag{S19}$$

Similarly, $\tilde{A}(-\Omega_s)$ can be derived as,

$$\tilde{A}\left(-\Omega_s\right)=\frac{i\delta\omega}{2\left[-i\Omega_s+\frac{\kappa}{2}+\frac{\delta\omega^2}{2(-i4\Omega_s+\kappa)}\right]}\tilde{A}(0). \tag{S20}$$

Hence, when $\delta\omega$ is relatively large, the $\delta\omega^2$ term cannot be neglected and causes nonlinear response for $\tilde{A}(\pm\Omega_s)$ and the output sensing signal. When it is small, Eqs. S19, S20 reduce to Eqs. S7, S8.

Strong sidebands of $\tilde{A}(\pm\Omega_s)$ can also cause depletion of the intracavity pump $\tilde{A}(0)$. When considering this depletion, $\tilde{A}(0)$ should be (see Eqs. S4, S5),

$$\tilde{A}(0)=\frac{i\delta\omega\left[\tilde{A}\left(-\Omega_s\right)+\tilde{A}\left(\Omega_s\right)\right]+i2\sqrt{\kappa_e/T_R}E_{\mathrm{in}}}{\kappa}. \tag{S21}$$

When combining Eqs. S19, S20 S21 to solve for $\tilde{A}(\pm\Omega_s)$, the pump depletion can further contribute to the nonlinear response of the MEFS and causing a limited dynamic range. The corresponding solutions for $\tilde{A}(\pm\Omega_s)$ and $V_{\mathrm{RF}}(t)$ are very bulky. To visualize the signal saturation from higher-order sidebands and pump depletion, we plot the normalized amplitude of $V_{\mathrm{RF}}(t)$ under different approximations in Fig. S2a. For Eq. S15, the response is linear versus

$\delta\omega$. When taking 2nd sidebands into account, but excluding pump depletion (substituting Eq. S5 into Eqs. S19, S20), the response exhibits nonlinearity with a large $\delta\omega$ (dashed line in Fig. S2a). When further including Eq. S21, the response deviates from Eq. S15 further for a large $\delta\omega$. In other words, the linear dynamic range for MEFS becomes limited.

To validate the analysis, we also plotted the simulated MEFS response in Fig. S2a. The simulated response was obtained by solving Eq. S3 numerically for $\tilde{A}(\pm\Omega_s)$ to derive the normalized amplitude for V_{RF}. For a weak electric field or a small $\delta\omega$, Eq. S15 overlap with the simulation results. It validates that Eq. S15 can be used for modeling MEFS effectively for weak applied electric fields. For a relatively large $\delta\omega$, the theoretical response with 2nd sidebands and pump depletion included also agrees well with the simulation. Therefore, we conclude that the 2nd sidebands and pump depletion results in the observed signal saturation and a limited dynamic range of our MEFSs.

From Eqs. S19, S20, we notice that the $\delta\omega^2$ term is less significant for a large Ω_s. Moreover, $\tilde{A}(\pm\Omega_s)$ becomes weaker for a large Ω_s. Hence, the pump depletion also becomes weaker for a large Ω_s (see Eq. S21). Consequently, the upper limit of the dynamic range can be extended with a higher sensing frequency Ω_s. To show this trend, we plot the normalized response with Ω_s=0.5κ and 0.05κ in Fig. S2b. When Ω_s increases, the MEFS response becomes weaker, but the deviation between Eq. S15 and the model with higher-order sidebands and pump depletion included also becomes smaller. Note that it does not necessarily mean a larger dynamic range, as the sensitivity and minimum detected electric field may decrease due to the decreased MEFS response.

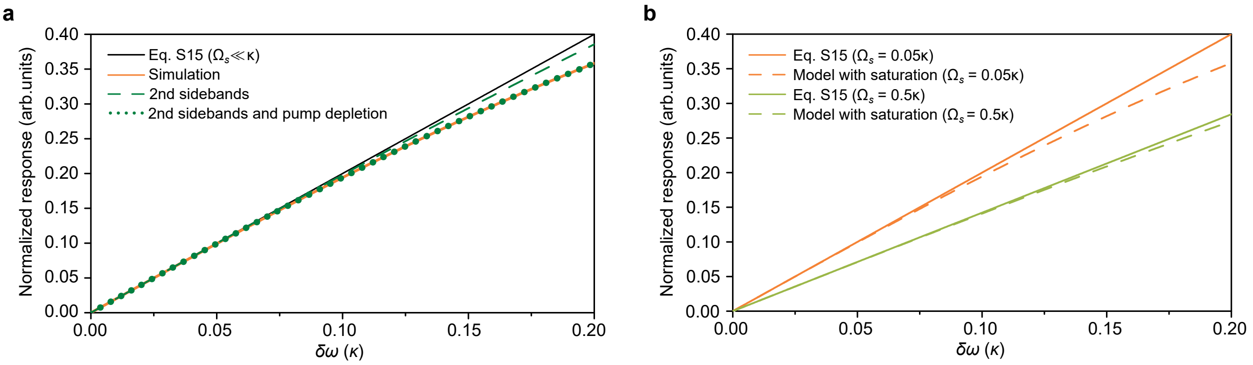

Fig. S2: **Saturation of sensing signal with strong electric fields.** **a**, Normalized amplitude of sensing signal $V_{\mathrm{RF}}(t)$ versus electric field induced resonance shift $\delta\omega$. The theory in Eq. S15 gives a linear response. When including the impacts from 2nd-order sidebands and pump depletion (see Eqs. S19, S20 and S21), theoretical analyses shows the sensing signal will saturate with a large $\delta\omega$. The theoretical analyses agree with the MEFS response obtained by numerically solving Eq. S3 for $\tilde{A}(\pm\Omega_s)$. The simulation also validates Eq. S15 (i.e., Eq. 1 in the main text) with a weak applied electric field. **b**, Normalized response obtained by Eq. S15 and the saturation model considering both second-order sidebands and pump depletion with $\Omega_s = 0.05\kappa$ and $\Omega_s = 0.5\kappa$. When the sensing frequency Ω_s is higher, the deviation of the sensing signal from Eq. S15 becomes smaller.

2. Noise floor of MEFS. The noise floor is another key factor determining the MEFS sensitivity. The minimum detectable electric field is defined as the signal with a signal-to-noise ratio (SNR) of 0 dB. The measured noise floor of Device 1 is shown in Fig. S3a (Device 2 shows similar noise floor). The noise floor rises in the range of 10 to 300 MHz due to the mixing between the relaxation oscillation of the ECDL around 2.9 GHz and $\Omega_m/2\pi$. By replacing the ECDL with a fiber laser, the bump vanished. In addition, the MEFS may suffer from other noise sources, including PD noise, laser relative intensity noise (RIN), shot noise and laser frequency noise.

1) Frequency noise. Frequency noise of the ECDL was measured by a self-heterodyne method [S2, S3]. In the interested frequency band, the laser was measured to exhibit a white frequency noise of $S_f(\omega)$=170 Hz^2/Hz. This frequency noise will contribute to a noise floor written as $S_f(\omega)S_{\mathrm{PDH}}^2/R_L$, where S_{PDH} is the PDH error signal slope and R_L=50 Ω is the load resistance of the electric spectrum analyzer. It yields a noise floor of -143.7 dBm/Hz (note that we used S_{PDH}=1.12 V/GHz or half of the value in the main text, as the oscilloscope had a loaded resistance of 1 MΩ).

2) Shot noise. Shot noise is calculated as $2\hbar\omega_l P_{\mathrm{PD}} K_{\mathrm{PD}}^2/R_{\mathrm{L}}$ [S4], where $\hbar$ is the reduced Planck constant, P_{PD} is PD input power (different from P_{in} in Eq. S15). This contribution was estimated to be -151 dBm/Hz with the conversion loss of the mixer included. This determines the fundamental limit of the noise floor for MEFSs.

3) PD noise and RIN. Figure S3a shows the noise floor is mainly contributed by the PD noise and RIN. To further quantify their contribution, we adjusted the input power for the PD and averaged the noise floor of Device

1 with the ECDL in the range of 300 MHz to 1 GHz (outside the relaxation oscillation related bump) in Fig. S3b. The measured floor increases quadratically (from laser RIN) with a constant floor (from PD). The quadratic fit shows the contribution from the PD is 3.3 dB stronger than the laser RIN when the PD input power is -10.7 dBm (used power in the measurement in the main text). Therefore, the SNR may further improve by increasing the input power. Nevertheless, the input power was limited by the ECDL power and the 10 dB fiber-to-fiber insertion loss in the experiment. If the input power is high enough (purple dashed line approaching the quadratic fit), the laser RIN will dominate over the PD noise and further input power increase will not contribute to SNR improvement. For strongly under-coupled or over-coupled microcavities, transmitted carrier may add to the noise floor but not the signal. Hence, critical coupling also contributes to higher sensitivity by lowering the noise floor.

Figure S3c shows a theoretical comparison between frequency noise induced noise floor and PD noise, by varying the loaded Q-factor (thus, S_{PDH}) and the laser frequency noise. When the Q-factor is high, low frequency noise is needed. Fortunately, ultralow frequency noise down to 1 $\mathrm{Hz}^2/\mathrm{Hz}$ has been possible for chip-based lasers [S5].

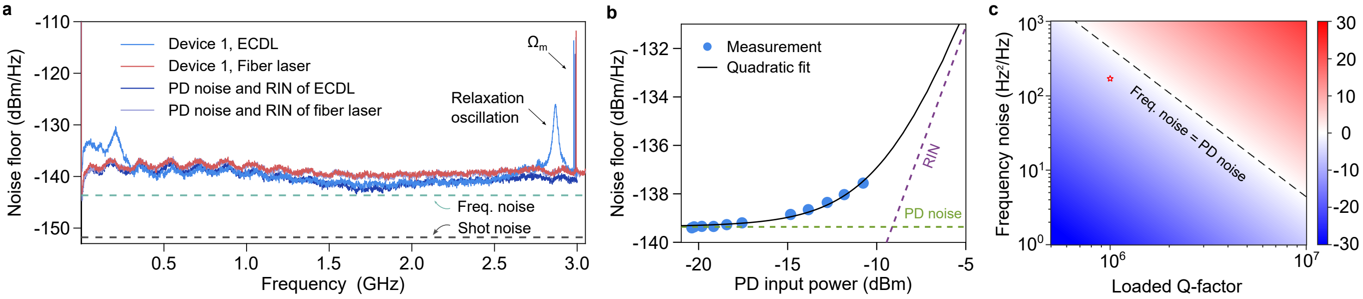

Fig. S3: **Noise floor of the MEFS.** **a**, Measured noise floor of Device 1 and contributions from different noise sources. **b**, Averaged noise floor for Device 1 in the frequency range of 300 MHz to 1 GHz under different PD input powers. The noise floor increases quadratically with increasing input power for the PD with a constant floor from the PD. **c**, The ratio between frequency noise induced noise floor and PD noise (in dB units) with different laser frequency noises and loaded Q-factors of the microcavities (thus the PDH error signal slope). The dashed line indicates a ratio of 0 dB. The red star denotes the condition for Device 1 in the main text.

3. Electric field gain of the antenna. We simulated the electric field gain from the MEFS antenna within the frequency range from 30 MHz to 10 GHz using the finite integration technique [S6]. The triangle dipole antenna has a dimension of 5 mm × 2 mm × 1 μm (length × bottom width × thickness), and is patterned on a silicon substrate. The field amplitude gain of the antenna, defined as the enhancement of the electric field amplitude between the electrodes over the applied electric field amplitude in the simulation, is shown in Fig. S4.

The resonance frequency of the antenna is 6.6 GHz. The field amplitude gain at 100 MHz (tested frequency in Fig. 3c in the main text) is 24.1 dB. In the frequency range from 80 MHz to 3 GHz, the simulated field amplitude gain varies by 1.2 dB (see the inset of Fig. S4). The measured MEFS response in power (amplitude) rolls off by 30 dB (15 dB) for Device 1 or by 20 dB (10 dB) for Device 2 in this frequency range (see Fig. 3d of the main text). Therefore, the antenna frequency response can be neglected when considering the MEFS bandwidth.

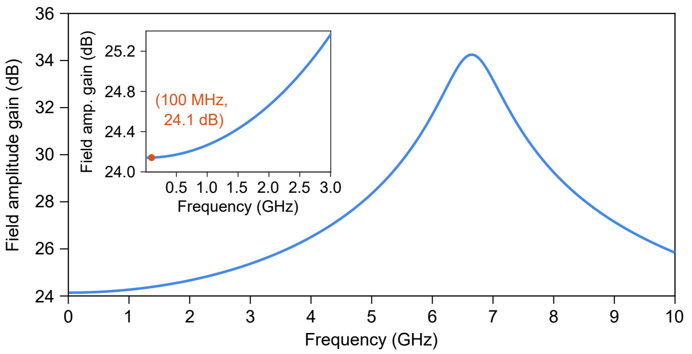

Fig. S4: **Simulated electric field amplitude gain of the antenna.** The antenna has a resonance frequency around 6 GHz. In the measured frequency range below 3 GHz, the antenna gain varies about 1.2 dB. The inset is a zoom in the frequency range from 30 MHz to 3 GHz. The orange point indicates the gain for 100 MHz, which is used frequency in Fig. 3c of the main text.

4. Detailed comparison with other electric field sensors. Detailed comparison with other electric field sensors on sensitivity, bandwidth and dynamic range are shown in Table S1. The corresponding figure can be found in Fig. 5a in the main text. The determination of the dynamic range involves measuring the maximum detectable electric field, which is defined as the electric field that corresponds to a sensor output deviating from linearity by 5% (the minimum detectable electric field is defined as in the main text).

Table S1: Detailed comparison with other electric field sensors

No.	Type	Sensitivity	Bandwidth	Dynamic range	Reference
1	Electrostatic force	$200\ \mathrm{V/(m\sqrt{Hz})}$	30 kHz	28 dB	[S7]
2	Electrostatic force	$173\ \mathrm{V/(m\sqrt{Hz})}$	300 Hz	55 dB	[S8]
3	Electrostatic force	$112\ \mathrm{V/(m\sqrt{Hz})}$	500 Hz	68 dB	[S9]
4	Electrostatic force	$172\ \mathrm{V/(m\sqrt{Hz})}$	1 kHz	72 dB	[S10]
5	Rydberg atom	$800\ \mu\mathrm{V/(m\sqrt{Hz})}$	50 kHz	80 dB	[S11]
6	Rydberg atom	$5.5\ \mu\mathrm{V/(m\sqrt{Hz})}$	100 kHz	90 dB	[S12]
7	Rydberg atom	$3.7\ \mathrm{mV/(m\sqrt{Hz})}$	30 MHz	65 dB	[S13]
8	Trapped-ion crystals	$77\ \mu\mathrm{V/(m\sqrt{Hz})}$	33 Hz	/	[S14]
9	Trapped-ion crystals	$240\ \mathrm{nV/(m\sqrt{Hz})}$	1 kHz	/	[S15]
10	NV center	$10\ \mathrm{V/(m\sqrt{Hz})}$	2 kHz	/	[S16]
11	NV center	$26\ \mathrm{kV/(m\sqrt{Hz})}$	1 MHz	/	[S17]
12	Induced charge	$380\ \mathrm{mV/(m\sqrt{Hz})}$	500 Hz	67 dB	[S18]
13	LN	$220\ \mu\mathrm{V/(m\sqrt{Hz})}^a$	300 MHz	100 dB	[S19]
14	LN	$294\ \mu\mathrm{V/(m\sqrt{Hz})}^a$	1.5 GHz	80 dB	[S20]
15	LN	$870\ \mu\mathrm{V/(m\sqrt{Hz})}$	35 MHz	>80 dB	[S21]
16	LN	$127\ \mathrm{mV/(m\sqrt{Hz})}$	18 GHz^b	58 dB	[S22]
17	LN	$20\ \mathrm{mV/(m\sqrt{Hz})}$	100 MHz^c	100 dB	[S23]
18	LN	$840\ \mathrm{V/(m\sqrt{Hz})}$	1 MHz	45 dB	[S24]
19	LN	$35\ \mathrm{mV/(m\sqrt{Hz})}$	26 GHz^b	117 dB^d	[S25]
20	TFLN	$7\ \mathrm{mV/(m\sqrt{Hz})}$	800 MHz	/	[S26]
21	TFLN	$4.5\ \mathrm{V/(m\sqrt{Hz})}$	1.86 GHz	/	[S27]
22	TFLN	$32\ \mathrm{V/(m\sqrt{Hz})}$	10 MHz^c	59 dB	[S28]
23	TFLN	$180\ \mathrm{mV/(m\sqrt{Hz})}$	750 MHz	/	[S29]
24	TFLN	$80\ \mathrm{mV/(m\sqrt{Hz})}$	2.5 GHz	/	[S29]
25	TFLN	$8.4\ \mathrm{mV/(m\sqrt{Hz})}$	100 MHz^c	91 dB	[S30]
26	TFLN	$8.8\ \mathrm{mV/(m\sqrt{Hz})}$	26 GHz^b	117 dB	[S31]
27	TFLN	$8.8\ \mu\mathrm{V/(m\sqrt{Hz})}$	110 MHz	123 dB	This work
28	TFLN	$29.5\ \mu\mathrm{V/(m\sqrt{Hz})}$	414 MHz	122 dB	This work
29	TFLN	$10.5\ \mu\mathrm{V/(m\sqrt{Hz})}$	101 MHz	122 dB	This work
30	TFLN	$15.5\ \mu\mathrm{V/(m\sqrt{Hz})}$	258 MHz	117 dB	This work
31	TFLN	$5.2\ \mu\mathrm{V/(m\sqrt{Hz})}$	116 MHz	/	This work, fiber laser

^{a}Receiving antenna with length up to 40-50 mm were used. ^{b}±10 dB deviation or 10 dB roll-off. ^{c}The sensing frequency measurement is limited to the range of the given data; larger bandwidth may be possible. ^{d}Dynamic range is not given by the paper and is estimated here.

[S1] Black, E. D. An introduction to pound–drever–hall laser frequency stabilization. *American Journal of Physics* **69**, 79–87 (2001).

[S2] Yuan, Z. *et al.* Correlated self-heterodyne method for ultra-low-noise laser linewidth measurements. *Optics Express* **30**, 25147–25161 (2022).

[S3] Liu, K. *et al.* Fundamental linewidth of an aln microcavity raman laser. *Optics Letters* **47**, 4295–4298 (2022).

[S4] Ye, J., Ma, L.-S. & Hall, J. L. Ultrasensitive detections in atomic and molecular physics: demonstration in molecular overtone spectroscopy. *Journal of the Optical Society of America B* **15**, 6–15 (1998).

[S5] Guo, J. *et al.* Chip-based laser with 1 Hertz integrated linewidth. *Science Advances* **8**, eabp9006 (2022).

[S6] Marklein, R. *The finite integration technique as a general tool to compute acoustic, electromagnetic, elastodynamic, and coupled wave fields* (IEEE Press and John Wiley and Sons, New York, NY, USA, 2002).

[S7] Zhu, T., Zhou, L., Liu, M., Zhang, J. & Shi, L. High sensitive space electric field sensing based on micro fiber interferometer with field force driven gold nanofilm. *Scientific reports* **5**, 1–7 (2015).
[S8] Kainz, A. *et al.* Distortion-free measurement of electric field strength with a mems sensor. *Nature electronics* **1**, 68–73 (2018).
[S9] Han, Z., Hu, J., Li, L. & He, J. Micro-cantilever electric field sensor driven by electrostatic force. *Engineering* (2022).
[S10] Han, Z., Xue, F., Hu, J. & He, J. Trampoline-shaped micro electric-field sensor for ac/dc high electric field measurement. *IEEE Transactions on Industrial Electronics* **69**, 13791–13798 (2021).
[S11] Sedlacek, J. A. *et al.* Microwave electrometry with rydberg atoms in a vapour cell using bright atomic resonances. *Nature Physics* **8**, 819–824 (2012).
[S12] Jing, M. *et al.* Atomic superheterodyne receiver based on microwave-dressed rydberg spectroscopy. *Nature Physics* **16**, 911–915 (2020).
[S13] Liu, B. *et al.* Highly sensitive measurement of a megahertz rf electric field with a rydberg-atom sensor. *Physical Review Applied* **18**, 014045 (2022).
[S14] Affolter, M., Gilmore, K., Jordan, J. & Bollinger, J. Phase-coherent sensing of the center-of-mass motion of trapped-ion crystals. *Physical Review A* **102**, 052609 (2020).
[S15] Gilmore, K. A. *et al.* Quantum-enhanced sensing of displacements and electric fields with two-dimensional trapped-ion crystals. *Science* **373**, 673–678 (2021).
[S16] Michl, J. *et al.* Robust and accurate electric field sensing with solid state spin ensembles. *Nano letters* **19**, 4904–4910 (2019).
[S17] Qiu, Z., Hamo, A., Vool, U., Zhou, T. X. & Yacoby, A. Nanoscale electric field imaging with an ambient scanning quantum sensor microscope. *npj Quantum Information* **8**, 107 (2022).
[S18] Ghionea, S. *et al.* Mems electric-field sensor with lead zirconate titanate (pzt)-actuated electrodes. In *SENSORS, 2013 IEEE*, 1–4 (IEEE, 2013).
[S19] Kuwabara, N., Tajima, K., Kobayashi, R. & Amemiya, F. Development and analysis of electric field sensor using linbo/sub 3/optical modulator. *IEEE transactions on electromagnetic compatibility* **34**, 391–396 (1992).
[S20] Schwerdt, M., Berger, J., Schuppert, B. & Petermann, K. Integrated optical e-field sensors with a balanced detection scheme. *IEEE transactions on electromagnetic compatibility* **39**, 386–390 (1997).
[S21] Hsu, R. C., Ayazi, A., Houshmand, B. & Jalali, B. All-dielectric photonic-assisted radio front-end technology. *Nature Photonics* **1**, 535–538 (2007).
[S22] Sun, B., Chen, F., Chen, K., Hu, Z. & Cao, Y. Integrated optical electric field sensor from 10 khz to 18 ghz. *IEEE Photonics Technology Letters* **24**, 1106–1108 (2012).
[S23] Toney, J. E., Pollick, A., Retz, J. & Sriram, S. Noncontact electro-optic near field probe for surface electric field profiling. In *2016 IEEE SENSORS*, 1–3 (IEEE, 2016).
[S24] Yang, Q., Sun, S., He, Y. & Han, R. Intense electric-field optical sensor for broad temperature-range applications based on a piecewise transfer function. *IEEE Transactions on Industrial Electronics* **66**, 1648–1656 (2018).
[S25] Zhang, J., Chen, F. & Liu, B. Integrated photonic electric field sensor operating more than 26 ghz. *IEEE Microwave and Wireless Components Letters* **30**, 1009–1012 (2020).
[S26] Toney, J. E. *et al.* Photonic crystal electro-optic devices in engineered thin film lithium niobate substrates. In *Photonic Microdevices/Microstructures for Sensing IV*, vol. 8376, 100–111 (SPIE, 2012).
[S27] Chen, L. & Reano, R. M. Compact electric field sensors based on indirect bonding of lithium niobate to silicon microrings. *Optics Express* **20**, 4032–4038 (2012).
[S28] Calero, V. *et al.* An ultra wideband-high spatial resolution-compact electric field sensor based on lab-on-fiber technology. *Scientific Reports* **9**, 1–10 (2019).
[S29] Toroghi, S. & Rabiei, P. Thin film lithium niobate electric field sensors. *Review of Scientific Instruments* **93**, 034702 (2022).
[S30] Xue, Y., Ruan, Z. & Liu, L. Electrode-free photonic electric field sensor on thin film lithium niobate with high sensitivity. *Opt. Lett.* **47**, 2097–2100 (2022).
[S31] Liu, Z. *et al.* Broadband electric field sensor with large dynamic range and high sensitivity based on lithium niobate on insulator. *IEEE Photonics Technology Letters* (2022).
